# How Stressful Is Maternity? Study about Cortisol and Dehydroepiandrosterone-Sulfate Coat and Claws Concentrations in Female Dogs from Mating to 60 Days Post-Partum

**DOI:** 10.3390/ani11061632

**Published:** 2021-05-31

**Authors:** Jasmine Fusi, Tanja Peric, Monica Probo, Alessio Cotticelli, Massimo Faustini, Maria Cristina Veronesi

**Affiliations:** 1 Department of Veterinary Medicine, Università degli Studi di Milano, via dell’Università, 6, 26900 Lodi, Italy; jasmine.fusi@unimi.it (J.F.); massimo.faustini@unimi.it (M.F.); maria.veronesi@unimi.it (M.C.V.); 2 Department of Agricultural, Food, Environmental and Animal Sciences, University of Udine, Via Sondrio, 2/a, 33100 Udine, Italy; tanja.peric@uniud.it (T.P.); alessio.cotticelli@uniud.it (A.C.)

**Keywords:** dog, maternity, cortisol, dehydroepiandrosterone-sulfate, HPA axis

## Abstract

**Simple Summary:**

Canine pregnancy and post-partum (total duration around 120 days) is a very intense period and could be a trigger for the activation of the Hypothalamic–Pituitary–Adrenal (HPA) axis in female dogs, given the higher allostatic load. To evaluate this activation, the concentrations of Cortisol (C) and Dehydroepiandrosterone-sulfate (DHEA-S), final products of the HPA axis, were detected in the coat and claws of 15 Dobermann Pinscher female dogs, with monthly collections from mating until the end of weaning. The C concentrations, both in the coat and claws, showed a significant trend of increase from mating until 60 days post-partum. In both matrices, the DHEA-S changes were not significant. Maternal parity and litter size did not play a significant influence on the concentrations of C and DHEA-S in both matrices. The results of the present study seem to depict maternity as a main activator of the HPA axis that, in turn, leads to the secretion of C. This is probably due to the increased allostatic load for the mothers, although it is not possible to discern the precise role of the multiple processes characterizing this period (uterine involution, lactation, nursing and grooming of the puppies).

**Abstract:**

In dogs, the phase from mating to the end of weaning lasts about 120 days and encompasses many aspects that, interacting, contribute to increase the allostatic load. The coat and claws, useful for long-term change assessments, have the advantage of being collectable without invasiveness. In the present study, the Cortisol (C) and Dehydroepiandrosterone-sulfate (DHEA-S) concentration monthly changes in the coat and claws were studied in female dogs from mating to the end of weaning to assess Hypothalamic–Pituitary–Adrenal (HPA) axis activation during pregnancy and the post-partum period. The results from 15 Dobermann Pinscher female dogs showed a trend of increase of the coat C from mating to 60 days post-partum, with significant changes between mating and parturition-60 days post-partum (*p* < 0.01) and between the 30-day pregnancy diagnosis (PD) and 30–60 days post-partum (*p* < 0.05). The claws C trend showed significant increases between mating and 30–60 days post-partum (*p* < 0.05) and between the PD and 60 days post-partum (*p* < 0.01). DHEA-S in both matrices showed non-significant changes. The results suggest that maternity could play a pivotal role in the HPA axis activation, with a subsequent chronic secretion of C determining an increase in the allostatic load in the mothers. Neither maternal parity nor litter size played a significant role in the accumulation of C and DHEA-S in both matrices.

## 1. Introduction

In contrast to horse and cow newborns, newborn dog puppies are considered altricial and strictly dependent on the mother to survive until the end of weaning [1]. Canine pregnancies last about 63 ± 1 days after ovulation [2,3], and, at parturition, the phase of lactation, nursing and puppies grooming begins. In breeding facilities, the weaning of puppies starts at about 30 days after parturition, ending at 60 days post-partum. In Italy, puppies are usually sold to the new owners not earlier than 60 days of age and usually between 60 and 65 days of age. Therefore, the time elapsing between mating of the female dog and the end of weaning lasts about 120 days: altogether, this phase is complex and encompasses reproductive, metabolic, emotional and behavioral processes, often interacting among them, in turn increasing the allostatic load of the animal. This long-term period deserves scientific interest, as it represents a challenging and possibly very stressful time for the female dog [1,4]. Although many studies have focused on the specific phase of pregnancy, parturition and post-partum, to the authors’ knowledge, the whole period from mating until the end of weaning has only rarely been investigated in dogs [5]. Nonetheless, like what happens in horse husbandry, the selection of breeding female dogs is not based on the actual reproductive aptitude but, more often, on their show performances and genetic value. Due to the important role of maternal aptitude in puppies rearing, a more accurate selection of breeding female dogs could ameliorate the quality of dog breeding [6], contributing to limit the still too-high percentages of perinatal mortality [7] and conveying more ethical dog breeding. Still, today, it is not uncommon to observe disturbed behavior in periparturient female dogs, possible aggressiveness towards the newborns or even cannibalism, often accompanied by insufficient grooming or nursing of the puppies, thus impairing the successful outcome of litters. Among the diverse causes underlying these troubles, environmental factors, genetics, parity and number of the newborns in a litter were suggested [1,4,6]. Other than that, the role of the Hypothalamic–Pituitary–Adrenal axis (HPA axis) in shaping maternal behavior was presumed in sheep [8,9]. A study on adrenalectomized rats reported that, when the primiparae were injected with high doses of corticosterone, they showed higher levels of maternal care (notably, licking behavior) than the other primiparous rats who received lower doses of corticosterone [9,10]. Additionally, in humans, the HPA axis was reported to exert a role in mother–infant bonding [11]. In gorillas, a correlation between maternal behavior, stress and urinary Cortisol (C) concentrations was found; higher C concentrations were found in stressed mothers, whose infants were often less-cared-for [12]. In dogs, a similar association was presumed; mothers experienced peaks of salivary C after being temporarily separated from their litters, with higher peaks in mothers who displayed the most the typical maternal care behaviors [13]. It is thus evident that the role of stress is of utmost importance when evaluating data about the experience of maternity, both in humans and animals.

Lactation, especially when associated with nursing and grooming, is a challenging physiologic phase that requires a great expenditure of energy and could represent a source of stress in relation to the litter size [14] and to parental efforts. The increased metabolic load and stress are associated with activation of the HPA axis, leading to an increase of circulating C [14]. In a recent study on female cats, reference [14] reported that the highest concentrations of C in the blood were found 4 weeks after parturition, in association with the peak of lactation, suggesting a major effect of lactation and kitten nursing on HPA axis activation. Those authors concluded that measuring the circulating C concentrations could be helpful in deepening the understanding of the reaction of queens through the physiological stresses occurring during lactation.

Other than C, dehydroepiandrosterone (DHEA) and its sulfated form, dehydroepiandrosterone sulfate DHEA-S, are also final products of the activation of the HPA axis. Among multiple actions, DHEA is notably reported to be a neuroactive steroid with antidepressant actions, associated with some types of behaviors like sex recognition and aggressiveness [15,16,17,18]. In the literature, some results about the concentrations of circulating DHEA and DHEA-S during pregnancy exist in women [19], cows [20] and, more recently, in killer whales [21]. However, the results are conflicting: in women, a decline of circulating DHEA and DHEA-S during pregnancy was reported; in killer whales, a rise of DHEA-S from early and during mid and late pregnancy in comparison to pre-fertilization and post-partum was detected; lastly, a significant increase of circulating DHEA throughout pregnancy was detected in heifers and cows [19,20,21].

However, the HPA axis could not be considered as a “closed system”, and its activity is modulated by factors such as oxytocin, able to exert an inhibitory action on the HPA axis. A recent study reported that the salivary oxytocin concentrations in female dogs were negatively correlated with excessive sniffing/poking behavior, considered as a possible sign of maternal distress associated with lactation [22]. Moreover, an allostatic theory of oxytocin was reported. According to this theory, the oxytocin system should be able to support the refinement of a physiologic setting to cope with adaptation and survival [23].

Despite most of the studies referring to concentrations of C and DHEA/DHEA-S in the blood or less invasive matrices such as urines, feces and saliva, all those matrices depict only the concentration of a given time point, and their use is therefore not suitable when long-lasting physiological processes have to be investigated because of the need to repeat multiple samplings. To overcome these issues, in the last years, new matrices, like the hair/coat and nails/claws, have been proposed for long-term hormonal studies in humans and animals [24,25,26,27,28] thanks to their characteristic of providing retrospective information about long-term hormone accumulation. Thus, the coat and claws could be suitable matrices for the study of hormonal changes occurring in the long-lasting phase between mating of the female dog to puppies weaning.

Therefore, the aim of the present study was to investigate the possible activation of the maternal HPA axis during the dynamic and challenging phase lasting from mating of the female dog to puppies weaning through the assessment of C and DHEA-S concentrations in the maternal coat and claws monthly collected.

## 2. Materials and Methods

### 2.1. Ethics

Although coat and claws sampling is a noninvasive procedure, this trial was carried out in accordance with EU Directive 2010/63/EU, and it was approved by the Ethical Committee of the University of Milan (OPBA_33_2021). Written informed consent was signed by the owners, giving permission to submit each female dog to elective C-section, to collect coat and claws samples and to allow the record of clinical data for research purposes.

### 2.2. Animals

Sixteen purebred Doberman Pinscher, 2nd to 3rd parity female dogs, aged 3–6 years old (mean ± SD: 4.1 ± 1.06), belonging to a single breeder were enrolled in the study. All the female dogs were found to be healthy by a clinical examination and by routine health tests performed by a veterinarian and submitted to the common vaccination and parasite prophylaxes, fed with commercial food and housed in a single kennel with indoor and outdoor spaces. The animals were always handled and managed by the same operator. All the female dogs showed a history of previous normal pregnancies, post-partum and lactation phases. In all of them, an elective cesarean section (ELCS) was performed at the previous whelping, because of the large litter sizes and the consequent risk for dystocia. Of the 16 mated female dogs enrolled in the present study, one was diagnosed as not pregnant at 30 days after ovulation and therefore removed from the study.

### 2.3. Estrus and Mating

From the onset of proestrus, all the female dogs were monitored through serial vaginal smears every 48–72 h and through the assay of plasma progesterone concentrations performed every 48 h from the beginning of the signs of cytological estrus. Based on these parameters, all the female dogs were submitted to a single mating, performed with a male of proven fertility, 48 h after the estimated ovulation, when progesterone plasma concentrations ranged between 4 and 10 ng/ml [29]. Each female dog was mated with a different healthy male of proven fertility. At the time of mating, the BCS of each female dog was assessed on a scale of 5.

### 2.4. Pregnancy, Caesarean Section, Newborn Puppies and Post-Partum Management

At 30 days after the estimated ovulation, pregnancy was checked by ultrasonographic examination, and the pregnant female dogs were submitted to measurement of the inner chorionic cavity (ICC) for the first calculation of the parturition date [30], while the nonpregnant female dogs were excluded from the study.

At 45 days after ovulation, a second ultrasonographic evaluation was performed to assess the normal course of pregnancy, the correct development and wellbeing of the fetuses and to measure the biparietal (BP) diameter for an additional calculation of the parturition date [30].

Given the reported higher risk for dystocia when the litter size counts more than 9 puppies [31], and because all the enrolled Dobermann Pinscher female dogs carried pregnancies with more than 9 fetuses, they were all scheduled for ELCS at term of pregnancy. The day for performing ELCS was scheduled on the basis of the concordance of several parameters, as previously reported [32,33,34,35,36]: date of ovulation, based on the measurement of plasma progesterone concentrations, ICC and BP. Moreover, during the last days of pregnancy, before the expected parturition date, the female dogs were checked daily to assess their clinical conditions and for ultrasonographic evaluations of the fetal wellbeing. Other than this, plasma progesterone concentrations were assessed to identify the pre-parturient decrease [37]. The ELCSs were performed only when plasma progesterone concentrations were <2 ng/ml.

For all the ELCSs, the same anesthetic and surgical protocols, aimed to minimize the possible negative effects on newborn viability and mothers’ wellbeing, were performed, as *previously* reported [32,35,36].

Two expert neonatologists took care of the newborns as soon as they were extracted from the uterus, providing professional assistance. Within 5 minutes after birth, newborn viability was assessed by an Apgar score, and the puppies were classified as viable when the Apgar score was ≥7 [38]. The newborns were also evaluated for the absence of gross physical malformations or defects.

According to the viability classification, all the subjects were submitted to routine neonatal care or different degrees of neonatal assistance, as previously reported [38]. Birthweight, gender and litter size were also recorded.

Mothers and litters were discharged when female dogs were awake and showed normal behavior toward the puppies and after having verified the presence of normal mammary secretions.

A daily follow-up to update the general conditions was provided by the breeder from the day after parturition. Maternal and litter clinical data were daily recorded, together with the puppies’ bodyweight until 60 days after parturition. In addition, at 1 and 2 weeks, and at 30 and 60 days after parturition, clinical examinations were scheduled. At 60 days after parturition, maternal BCS was reassessed.

### 2.5. Samples Collection

Coat samples were collected by using a razor (TN2300 Nomad, Rowenta^®^ spa, Milan, Italy), allowing to shave an area of about 5 cm^2^ from the dorsal surface of the right forearm until the level of the skin (coat samples). The tips of the claws were collected from all the digits of both forearms with a claw clipper (C135, Candure^®^ Services LTD, Leicester, UK) and stored (claws samples). Coat and claws samples were placed in separate paper envelopes, labeled with a univocal code and stored at room temperature until the analysis.

The first sample collection was performed the day of mating. At the time of the pregnancy diagnosis, the second coat and claws sampling was performed, collecting only the regrown area.

At parturition, the third sample collection was performed. This collection did not always respect the 30 days of interval previously scheduled for the other samplings. Given that parturition occurs at about 60–62 days after ovulation, indeed, the interval of time between the second sampling and the one at parturition could have been 30 + a few days.

In all the cases, before anesthesia induction, the regrown coat and claws were collected and stored as reported above.

At 30 and 60 days post-partum, the regrown coat and claws were further collected and stored as reported above.

At every sampling time, after the collection, the razor and the claw clipper were disinfected with a 70% alcohol solution [39].

### 2.6. Hormone Analysis

Coat strands and claws were washed in 3-mL isopropanol to ensure the removal of any steroids on their surface. Coat and claws steroids were extracted with methanol and measured by radioimmunoassay (RIA). The concentrations of Cortisol and Dehydroepiandrosterone sulphate (DHEA-S) were measured using a solid-phase microtiter RIA. In brief, a 96-well microtiter plate (OptiPlate; PerkinElmer Life Sciences Inc., Zaventem, Belgium) was coated with goat anti-rabbit γ-globulin serum diluted 1:1.000 in 0.15-mM sodium acetate buffer (pH 9) and incubated overnight at 4 °C. The plate was then washed twice with RIA buffer (pH 7.5) and incubated overnight at 4 °C with 200 μL of the antibody serum diluted 1:20,000 for Cortisol and1:800 for DHEA-S. The cross-reactivities of the anti-Cortisol antibody with other steroids were as follows: Cortisol 100%, cortisone 4.3%, corticosterone 2.8%, 11-deoxycorticosterone 0.7%, 17-hydroxyprogesterone 0.6%, dexamethasone 0.1%, progesterone, 17-hydroxypregnenolone, DHEA-S, androsterone sulphate and pregnenolone < 0.01%. The cross-reactivities of the anti-DHEA-S antibody with other steroids were as follows: DHEA-S, 100%; androstenedione, 0.2%; DHEA, <0.01%; androsterone, <0.01% and testosterone, <0.01%. After washing the plate with RIA buffer, the standards (5–200 pg/well), the quality control extract, the test extracts and the tracer (hydrocortisone {Cortisol [1,2,6,7-3H (N)]-}, DHEA-S [1,2,6,7-3H (N)] were added, and the plate was incubated overnight at 4 °C. The bound hormone was separated from the free hormone by decanting and washing the wells in RIA buffer. After the addition of 200 μL of scintillation cocktail, the plate was counted on a β-counter (Top-Count; PerkinElmer Life Sciences Inc.).

The intra- and inter-assay coefficients of variation were 3.7 and 10.1% and 3.2 and 11.8% for Cortisol and DHEA-S, respectively. The sensitivities of the assays were 1.23 pg/well and 0.54 pg/well for Cortisol and DHEA-S, respectively.

### 2.7. Statistical Analysis

A Shapiro–Wilk test was used to verify the normal distribution of data, followed by an ANOVA and post-hoc test, to assess the possible effects of the sampling time, litter size, maternal age and parity on the C and DHEA-S concentrations in the coat and claw samples. Statistical significance was set for *p* < 0.05 (JASP^®^, ver. 9 for Windows platform). The Pearson correlation test was used to assess the possible correlations between the two matrices for each hormone.

## 3. Results

### 3.1. Clinical Findings

The 15 pregnant female dogs (BCS at mating, mean ± SD: 3.0 ± 0.00) showed normal courses of pregnancy and, because all of them carried more than nine fetuses, were submitted to the ELCS at 60–63 (mean ± SD: 61.2 ± 1.09) days after ovulation, providing a total of 15 litters with a total of 163 puppies (three stillborn). The litter sizes ranged between 10 and 13, with a mean ± SD of 10.9 ± 1.13. Seventy-five males and 85 females, mean ± SD birthweight: 427.7 ± 87.82 g, mean ± SD Apgar score: 8.9 ± 0.66, were enrolled. Consequently, all the 15 female dogs were followed for the whole period elapsing from mating until the end of weaning at 60 days post-partum, when the BCS was 2.5 ± 0.24 (mean ± SD).

### 3.2. Coat and Claws C and DHEA-S Concentrations

**Figure 1:**
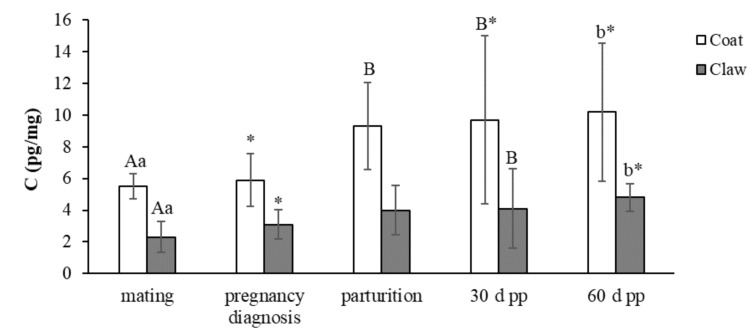
Concentrations (mean ± SD) of C in the coats and claws from mating to 60 days post-partum in the 15 female dogs. ^A,B^ *p* < 0.05; ^a,b^ *p* < 0.01 denote within-row significance. * *p* < 0.05 denotes within-row significance.

**Figure 2:**
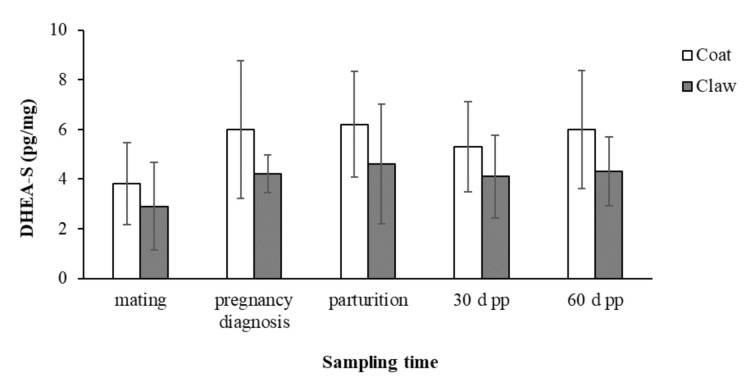
Concentrations (mean ± SD) of DHEA-S in the coats and claws from mating to 60 days post-partum in the 15 female dogs.

The concentrations (mean ± SD) of C and DHEA-S in the coat and claws from mating to 60 days post-partum in the 15 female dogs enrolled in the study are reported in Figure 1 and Figure 2, respectively.

The concentrations of C in the coats showed a trend of increase from mating to 60 days post-partum, with significant changes between mating and parturition-60 days post-partum and between the pregnancy diagnosis and 30–60 days post-partum. In the claws, the trend of C was very similar to the one observed in the coats, with significant increases between mating and 30–60 days post-partum and between pregnancy diagnosis and 60 days post-partum.

The Pearson correlation test showed a significant positive correlation (r = 0.277; *p* < 0.05) between the coat and claw C concentrations.

The concentrations of DHEA-S in both the coats and claws showed the same trend of increase between mating and all the subsequent sampling times but without significant changes.

The statistical analysis showed the absence of significant effects of the maternal age and litter size on the C and DHEA-S concentrations in the coats and claws, and no significant correlations of DHEA-S between the two matrices was found.

## 4. Discussion

To the authors’ knowledge, this was the first study reporting the concentrations of C and DHEA-S- in the coat and claws of female dogs from mating to 60 days post-partum, providing new information about long-term hormonal changes during gestation and the post-partum period in dogs, a topic that needs to be further investigated also in humans [40].

Some studies previously demonstrated the usefulness of a single coat sample for the study of C [25] and DHEA-S [41] concentrations in dogs. However, the present study offered the first evidence that, using the shave–re-shave method [27,42,43,44] after the first sampling, it is possible to collect only the regrown matrices, providing an optimal tool to investigate the long-term hormonal variations in dogs, as previously reported for other species [45,46,47]. However, in valuable dogs, the owners often refuse to shave the hair, even in a small area, limiting the use of the coat as a matrix of study in many instances. A couple of recent studies [26,28] showed the usefulness of the claws as an alternative to the coat for the same long-term studies in newborn puppies. For these reasons, in the present study, both the coat and the claws were collected with the shave—re-shave and clip—re-clip method for the longitudinal analysis of C and DHEA-S long-term changes.

The 30-day collection interval was designed to address several targets. Firstly, it reduced, at the minimum, the number of samplings to restrict the possible disturbances for the pregnant/lactating female dogs. Secondly, this timing fits with the most important milestones of the reproductive process management in female dogs. Thirdly, it allowed the regrowth of a suitable amount of sample for the analysis. Fourthly, it was reasonable for identifying and studying long-term hormonal changes. About the area of the body chosen for the coat collection, the right forearm was used because it is shaved for blood collection and other clinical purposes.

About the enrollment criteria of the female dogs, in the present study, only one breed was selected to avoid possible genetic differences in HPA axis activation [48]. However, even within the same breed, some individual peculiarities in HPA axis activation could be presumed. Moreover, to avoid possible differences in the managerial, nutritional and environmental factors that could influence HPA axis activation, all the female dogs belonged to the same breeder and were managed by the same operator. Other than this, selecting a single breed and the consequent homogeneous litter size, together with similar parity, allowed an additional reduction of the possible variables in HPA axis activation. The choice of enrolling only female dogs of second and third parity was based on the hypothesized possible influence of the maternal experience in HPA axis activation, as reported for maternal behavior [6]. The exclusive inclusion of female dogs scheduled for ELCS, in addition, concurred to reduce the other possible variables related to the effects of different types of delivery on HPA axis activation, as previously observed [49].

About C, the trend of increase from mating to 60 days post-partum observed in both the coat and claws suggested a gradual activation of the maternal HPA axis during the delicate phase of pregnancy and post-partum in female dogs. The finding of the highest concentration of C at 60 days post-partum is intriguing and could be related to the complex interaction of multiple factors, such as uterine involution, lactation and, also, nursing and grooming of the puppies, therefore addressing not only metabolic and physiologic phenomena but, also, emotional and behavioral changes for female dogs. All these factors, in turn, play a role in the increase of the allostatic load for female dogs. Although it is not possible to discern the single contribution of each one of these processes on the activation of the HPA axis, it is possible to suppose that lactation and care of the puppies could promote a continuous stimulation on the HPA axis, leading to a chronic secretion of C, as evidenced by its long-term, retrospective accumulation in the coat and claws. In the present study, all the female dogs displayed normal maternal behavior in the present and previous pregnancies, so that the observed HPA axis activation could be supposed to reflect a “normal” process related to maternity in dogs. However, it is not possible to exclude that the energy expenditure needed for all of the processes above seen could stimulate C secretion in order to mobilize the depots, as supposed in reference [14], given the higher concentrations reported at the peak of lactation.

The statistical analysis did not show an effect of maternal parity on C coat and claw concentrations. This could be due to very limited differences in the parity of the enrolled female dogs. However, in the bottlenose dolphin, the profiles of C concentrations during pregnancy were not affected by age or parity [50], and the same results were reported for killer whales [21]. In a recent study, the authors of reference [14] reported that the maternal experience did not represent an influencing factor on C concentrations in the blood of queens at 4 weeks after parturition, while it was influenced by the size of the litter (higher in queens with up to three kittens than in queens with more than three kittens). In the present study, the litter size did not influence the C concentrations in the coats and claws. It should be noted that, in the enrolled female dogs, no extreme litter sizes were observed, and the litter sizes were rather homogeneous, with the number of puppies per litter ranging between 10 and 13. Moreover, about the possible effect played by the puppies on the maternal HPA axis activation, in the present study, excluding the three stillborn, the puppies were all healthy and viable at birth and for all the subsequent periods of observation, showing normal growth and development in the first 60 days of age.

Therefore, taking all these considerations together, the results of the present study should be considered representative of the “normal” conditions of both mothers and litters. Hence, it could be interesting to verify the possible differences of long-term maternal HPA axis activation according to breed, first parity, extreme litters, pathological situations of mothers and/or puppies, etc.

Beside the C concentrations in the coats and claws, it was unforeseen to observe no significant changes in the concentrations of DHEA-S in both matrices. Even if conflicting results are reported in women, cows and killer whales, the role of DHEA as a neuroactive steroid is known to be involved in behavioral changes [15,16,17,18], in stress responses [15,16,17] and in the stimulation of lobuloalveolar mammary gland development in rats [51]. Like C, the concentrations of DHEA-S in the coat and claws were not influenced by the maternal parity or litter size.

In a study by Fusi and colleagues [26], both the C and DHEA(S) concentrations measured in the claws collected from puppies since birth to 60 days of age were found to be higher in claws collected at birth than in the subsequent sampling times, with a trend of decreasing. Therefore, because no differences related to DHEA-S claw concentrations were found in the present study in female dogs, it could be supposed that DHEA-S is mainly synthesized by the fetus/newborn. This hypothesis could also be reinforced by the extremely different concentrations found in the present study in the claws of female dogs compared to the study in reference [26] in the claws of puppies when the same time from parturition to 60 days post-partum was concerned. In fact, in the study from reference [26], the DHEA(S) claw concentrations were about 50-fold higher in puppies at parturition, about 20-fold higher at 30 days post-partum and about 15-fold higher at 60 days post-partum, as compared to the female dogs investigated in the present study.

Additionally, about C, higher concentrations were found in the puppies of the same study when compared to the concentrations found in the female dogs of the present one, especially at parturition (about six-fold).

These comparisons seem to suggest that, in dogs, the fetal HPA axis activation plays an important role, especially around the time of parturition.

The results of the present study offer new insights in the role of maternity on the allostatic load of female dogs. This topic is of maximum importance, given the influence of the allostatic load on the health of an organism, as reported in humans [52], and suggests that, although it is a physiologic event, the periods of pregnancy and, especially, post-partum should be considered as possible stressors for female dogs.

## 5. Conclusions

In conclusion, the higher concentration of C in the coat and claws found at 60 days post-partum than those found at mating seems to suggest that a complex interplay among the physiologic, emotional and behavioral events in the post-partum period occurs in female dogs. Maternity seems to play a pivotal role in the HPA axis activation in female dogs, with a subsequent chronic secretion of C and long-term accumulation in the coat and claws. No significant differences were found in the DHEA-S concentrations in the different sampling times, and neither maternal parity nor litter size played a significant role in the accumulation of C and DHEA-S in both matrices.

## Data Availability

The data are available upon request from the authors.
